# Sync fast and solve things—best practices for responsible digital health

**DOI:** 10.1038/s41746-024-01105-9

**Published:** 2024-05-04

**Authors:** Constantin Landers, Alessandro Blasimme, Effy Vayena

**Affiliations:** Health Ethics and Policy Lab, ETH Zurich, Switzerland

**Keywords:** Health policy, Technology, Translational research, Medical ethics, Public health

## Abstract

Digital health innovation is expected to transform healthcare, but it also generates ethical and societal concerns, such as privacy risks, and biases that can compound existing health inequalities. While such concerns are widely recognized, existing regulatory principles, oversight methods and ethical frameworks seem out of sync with digital health innovation. New governance and innovation best practices are thus needed to bring such principles to bear with the reality of business, innovation, and regulation.

To grant practical insight into best practices for responsible digital health innovation, we conducted a qualitative study based on an interactive engagement methodology. We engaged key stakeholders (*n* = 46) operating at the translational frontier of digital health. This approach allowed us to identify three clusters of governance and innovation best practices in digital health innovation: i) inclusive co-creation, ii) responsive regulation, and iii) value-driven innovation. Our study shows that realizing responsible digital health requires diverse stakeholders’ commitment to adapt innovation and regulation practices, embracing co-creation as the default modus operandi for digital health development. We describe these collaborative practices and show how they can ensure that innovation is neither slowed by overregulation, nor leads to unethical outcomes.

## Introduction

Digital health is a rapidly advancing field expected to transform healthcare through digital technologies such as mobile applications and artificial intelligence^1–3^. However, ethical concerns such as privacy, fairness, and autonomy have been prominently discussed in relation to digital health^3,4^. Commentators and stakeholders have thus called for responsible digital health innovation. Responsible digital health innovation has been defined as “any intentional systematic effort designed to increase the likelihood of a digital health technology developed through ethical decision making, being socially responsible and aligned with the values and well-being of those impacted by it”^2,5^. International organizations and multinational companies have put forward and committed to a plethora of principles and guidelines for innovative approaches in digital health and AI^6^. However, the adoption of responsible digital health principles in practice lags far behind their almost universal acceptance^2,3,7^.

Several impediments stand in the way of efficient and responsible digital health innovation. For instance, stakeholder collaboration is ineffective due to missing incentives, a highly fragmented stakeholder landscape, and a lack of established collaboration practices - factors that are compounded by the dynamism of digital health innovation. Digital health innovators, in particular start-ups, lack ethical awareness due to insufficient training. Where awareness exists, innovators’ lack of core resources, such as access to collaborators or funding, can impede societally beneficial innovation. Complex, contradictory, or insufficient regulation fails to provide orientation for innovators, while regulators often lack digital health-specific instruments to establish adequate control mechanisms. As a result, regulatory and ethical frameworks for responsible innovation are increasingly out of sync with progress in digital health^8^.

Recent literature on digital health has begun to address some of these impediment by calling for new modes of regulation^9,10^, design-thinking that considers users’ concerns and incorporates ethical considerations, or even re-structuring innovators’ organizational model to enable them to drive responsible innovation^9,11,12^. Technical solutions to further responsible digital health innovation have also emerged^13^. However, these tools and approaches have not achieved broad adoption and it remains questionable whether they correctly and sufficiently address practical impediments to responsible innovation^14–16^. Most approaches, for instance, only focus on parts of the complex stakeholder ecosystem and innovation process, while power and responsibility are disseminated across the ecosystem^2,17^. The discussion on responsible digital health would benefit from a deeper exploration of what innovation and governance best practices key stakeholders consider as adequate to resolve impediments to digital health.

We have collected and analyzed insights from 46 digital health stakeholders in Switzerland through the ECOUTER methodology, a participatory form of qualitative research^18^. We used this methodology to encourage research participants to share hard-to-access practical knowledge and perspectives by collectively editing an online mind-map populated with over 1500 individual inputs on the part of engaged participants. Further details about our study protocol can be found in the methods section (see Fig. 5). This paper sets out to present stakeholder-driven insights on governance and innovation best practices that can channel digital health innovation towards socially desirable and ethically robust outcomes. As such, our work fills an important gap in the literature as it helps the field move closer to practically addressing much discussed ethical and societal implications of digital health.

## Results

Our data highlights the need for stakeholders in digital health (DH) to adapt specific governance and innovation best practices to meet ethical and societal goals: 1) inclusive co-creation, 2) responsive regulation, and 3) value-driven innovation (see overview in Table 1). Before describing governance best practices, participants provided a detailed account of leading digital health stakeholders detailed in Table 2.

**Table 1:** Overview of governance best practices for digital health innovation

	Inclusive co-creation	Responsive regulation	Value-driven innovation
Description	Diverse stakeholders co-create innovation throughout phases, following an aligned vision	Early exchanges, capability building, and sandboxing enable dynamic responses to innovation	Training, technology, and new business models enable innovators to drive responsible innovation
Lead actors	All stakeholders	Regulators	Innovators

**Table 2:** Digital health stakeholders

Stakeholder archetype^a^	Description and main activity
**Innovators**	Develop and market novel (typically technological) innovations in digital health and medical AI
Healthcare incumbents	Companies with existing healthcare products and services offerings (e.g., drugs, medical device, health insurance); typically integrate or bundle digital services with their existing products (e.g., mobile apps for drug compliance, remote monitoring software) or develop novel digital offerings
Digital disruptors	Companies with no major prior healthcare exposure, driving digital innovation in health. Major subcategories are start-ups and established “BigTech” companies (e.g., Microsoft, Apple); participants considered digital disruptors to have more technology, less healthcare-specifc capabilities than healthcare incumbents; they were also considered to be less compliance-oriented than the incumbents
Academic innovators	Research institutions advancing digital healthcare solutions; frequently lead to spin-offs (i.e., start-ups)
Innovation enablers	Provide vital resources to innovators, in particular start-ups and smaller firms; innovation enablers differ by the (major) support they provide, ranging from financing (VC investors, public funders), to providing space, facilities, contacts and training (innovation hubs and parcs); some enablers (esp. VC funds) retain a supervisory role in the innovator
**Regulators**	Develop legislation and policy; supervise innovators and enforce legislation
Law enforcement agency	Review regulatory applications (typically for medical device classification) and grant market access; monitor compliance with legistlation and standards
Notified bodies	Validate and certify medical device’s compliance with legislation and quality standards; prior to the EU’s 2017 MDR and IVDR, notified bodies advised innovators throughout the innovation process
Policymaker	Create and enforce policies; allocate public funding; occasionally guide or instruct law enforcement
Lawmakers	Draft, adopt, vote, and enforce legislation (i.e., parliament)
International organizations	Develop guidance, policy (e.g., WHO), and standards (e.g., IEEE, ISO)at an international level
Payors	Review and reimburse expenses for digital health tools and digitally enabled care; evaluate, grant reimbursement contracts for novel innovations (especially for supplementary insurance)
**Implementors & end-users**	Implementors adopt digital care tools and recommend tools to patients, end users use digital health tools
Patients	Use digital health tools directly or receive digitally enabled or augmented care; growing awareness and outspokenness of moral rights around digital health ethics; oftentimes represented by patient representative bodies
Citizens	Use and receive digitally enabled prevention; fund healthcare through taxes and insurance premiums; caretakers of relatives
Healthcare practitioners (HCPs)	Recommend and guide patients to use digital health tools (e.g., digital therapeutics); adopt digital tools and systems to enhance care provision (e.g., diagnosis, patient monitoring)
Hospitals	Adopt digitally enabled care tools and services; incentivize HCPs to adopt and recommend digital health tools

^a^Stakeholder archetypes are not exclusive. Several hybrid stakeholders (e.g., payors, health care practitioners) perform diverse roles in the innovation eco-system (e.g., develop, regulate, provide and use digital health innovation). Archetypes displayed in bold represent main stakeholder type, while stakeholder sub-categories belonging to these groups are displayed in normal font.

### Ethical issues of digital medical products

**Fig. 1:**
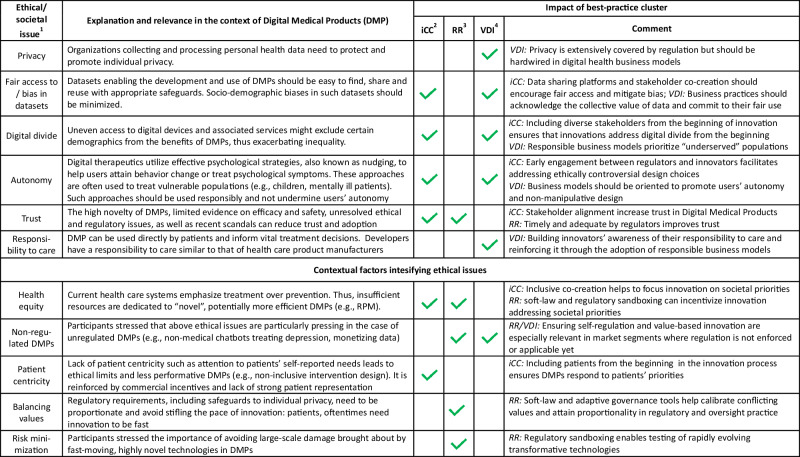
Ethical issues in DMPs mapped against identified best practices. Overview of core ethical issues in DMPs mapped against the best practices identified in this paper. The mapping highlights which best practices can help to address the specific ethical issues.

Participants also identified ethical issues, societal concerns, and perceived obstacles in digital health on a different section of the mind-map and connected them to the identified best practices (Fig. 1).

### Inclusive co-creation

**Fig. 2:**
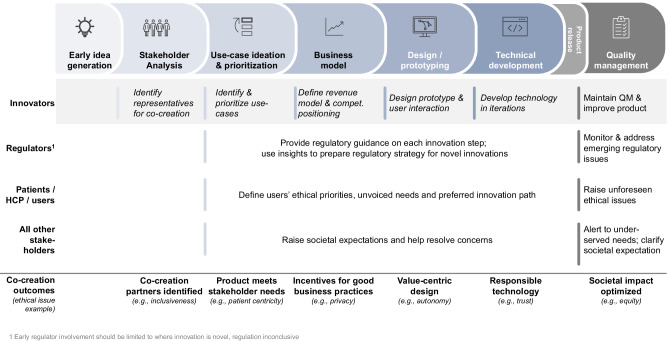
Overview of co-creation activities. Overview of how inclusive co-creation activities could occur along major phases of digital health innovation. This chart is illustrative – the types and sequence of innovation phases, actual co-creation activities, as well as stakeholder roles may vary across different DMPs.

Stakeholders concurred that regulators, patients, and citizens should be active co-creators throughout the digital health innovation process. Inclusive co-creation ensures that ethical and societal issues in digital health innovations, such as lack of patient centricity, trust, and autonomy, can be addressed (see Fig. 1). Such co-creation should be extensive and continuous covering activities such as defining the business model and commercial strategy of a novel technology (see Fig. 2). Participants stressed that stakeholders should also be actively involved in the technological development stage, helping to prioritize key design features (e.g., when to interact with patients) and resolving ethical issues (e.g., how to store and process data). Inclusive co-creation ensures that innovation prioritizes stakeholder concerns, addresses overlooked patient problems, and prevents harmful ideas at the design stage.

#### Aligned vision

Participants also recommended and discussed practical approaches to enable stakeholder co-creation. They agreed that developing an aligned vision for digital health would be highly beneficial for adopting co-creation. Such a vision should guide all core stakeholders – regulators, innovating companies, and even patients. An aligned vision should state the priorities, ethical values, and societal objectives of digital health innovation. As such, an aligned vision would also help to address public health priorities and make these choices explicit, as detailed in Fig. 2. Participants further stressed that an aligned vision should be as concrete as possible, enabling stakeholders to implement, track, and coordinate activities. To this end, defining a shared roadmap and quantifiable targets (e.g., key performance indicators) can enhance mutual understanding of different stakeholders’ values, priorities, and semantics, increasing trust and decreasing risk of future conflict. Formulating and following an aligned vision would thus further address ethical issues, such as building trust, or ensuring that patient needs are addressed.

#### Standards and platforms

To enable co-creation, participants further highlighted key enabling factors of successful co-creation such as secure data-sharing platforms to facilitate data availability by homogenizing data standards across industries (e.g., healthcare, cloud computing, app development). Participants also emphasized the role of “neutral” parties: universities, for example, were frequently suggested as potential intermediaries or trust brokers for collaboration between start-ups and pharma companies. Standards and platforms were also seen as a potent mechanism to further address ethical and societal concerns: integrating values such as equal access, autonomy, or patient centricity, standards and platform rules can enable stakeholders to address ethical and societal priorities.

### Responsive regulation

**Fig. 3:**
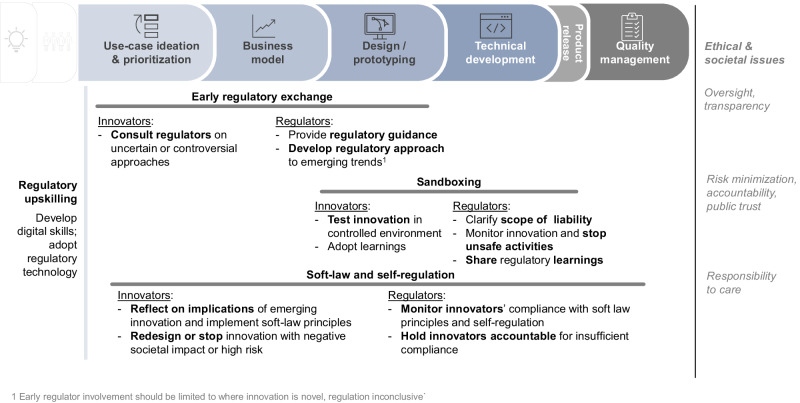
Overview of responsive regulation elements. Core elements and activities of responsive regulation along the major phases of digital health innovation. This chart is illustrative—the types and sequence of innovation phases, actual co-creation activities, as well as stakeholder roles may vary across different DMPs.

Regulators and innovators reported potential knowledge gaps as impediments to effective co-creation, amplifying ethical and societal risks. Regulators, for instance, may lack insights into the most recent technological trends. As a result, they may struggle to catch up and develop appropriate regulatory responses before highly disruptive innovations are launched. Where this happens, ethical and societal issues are likely to arise and remain unaddressed, especially if innovators lack sufficient awareness and adequate incentives to address these issues (c.f. Value Driven Innovation below). Participants also pointed out that high regulatory complexity leads innovators to—unknowingly or deliberately—avoid classifying their products and services as medical devices, which in turn would require them to comply with medical device regulations. As such, participants stressed that responsive regulation could help to address the ethical, societal, and regulatory issues of both, regulated and non-regulated medical devices (further detailed in Fig. 1), by providing regulatory clarity. Participants suggested several elements and processes to facilitate responsive regulation (shown in Fig. 3).

#### Early regulatory exchange

Early regulatory exchange consists of early, informal exchanges between innovators and regulators, and it is particularly important for disruptive innovations. As shown in Fig. 3, such exchange can occur from the early stages of innovation, long before regulatory applications are typically submitted via conventional regulatory approval processes. Participants perceived early regulatory exchange as an effective tool to prevent and address ethical and societal issues. Through early regulatory exchange, regulators can offer guidance from the very early stages of product development. In this capacity, they can alert innovators to issues, such as bias or privacy. Participants further suggested that such an approach could help innovators reduce inefficiencies that result from navigating inapplicable regulatory frameworks and costly re-designs that are often required after innovations fail to gain regulatory approvals at advanced implementation stages. Engaging regulators early in the innovation process, in turn, enhance regulatory learning and provides them time to identify ethical issues or react to contain fast-scaling risks (e.g., large-scale privacy breaches, malfunctioning diagnosis algorithms).

#### Soft law and self-regulation

Use of soft law instruments and self-regulation in contexts where the trajectory of digital health technology is highly dynamic and uncertain, and no specific legislation (“hard law”) exists yet. Soft law refers to non-binding instruments, agreements, or principles that do not have the same legal force as laws and regulations but can still influence behavior and shape norms and practices. Participants discussed the Swiss Code of Best Practice for Corporate Governance or the Federal “Experimentation Framework” for healthcare as examples of soft law (Art. 59b of Swiss E-KVG law)^19,20^.

Participants stressed that soft law requires higher self-regulation on behalf of innovators, who need to monitor and adapt their business practices accordingly. Participants stressed the practical importance of—at least partially—relying on and empowering soft law with reference to the high number of non-regulated digital health products. Participants pointed out that it would far exceed regulatory capabilities to regulate all non-regulated products that would qualify for regulation. Here, self-regulation shifts some of the burden from regulators, by requiring innovators to self-regulate, while regulators still need to monitor overall compliance with principles and legislation. Adoption of these tools holds promise to further protect and advance ethical and societal values, especially where traditional regulatory responses are insufficient.

#### Regulatory sandboxing

Participants suggested regulatory sandboxing as a system to further collaboration and learning between regulators and innovators in contexts where innovation is highly novel, and the applicability of existing laws and regulatory responses is uncertain. Before launching novel technologies, regulatory sandboxing allows innovators to test their innovation under close regulatory supervision. Regulators can simultaneously experiment with novel regulatory approaches to such innovation. Participants did, however, highlight that sandboxes should be focused on contexts where the innovation and regulation environment are uncertain.

Regulator capabilities must be updated regularly to ensure that regulation and regulatory enforcement are up to speed with digital health innovation. Participants also stressed the potential role of technology (such as machine learning analysis of submitted data sets) to significantly free up human regulatory resources and speed-up regulatory reviews.

### Value-driven innovation

**Fig. 4:**
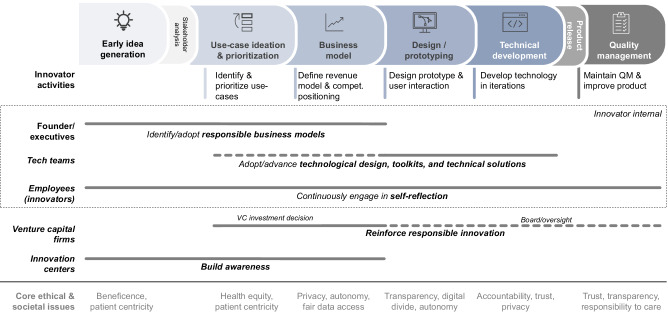
Overview of value-driven innovation activities. Overview of how different actors can advance elements and activities of value-driven innovation throughout the phases of digital health innovation.

Participants emphasized innovators’ considerable influence and responsibility in addressing ethical issues in digital health, for instance by adopting value-driven innovation practices. To this end, they recommended hardwiring ethical commitments and societal priorities into innovation processes and business models in digital health. Concretely, innovators should understand the ethical and societal implications of their activities, incorporate stakeholder perspectives, and strive to precisely define how their business model and products create benefit to users and society at large. As detailed in Fig. 4, several best practices for innovators and external actors can reinforce value-driven innovation, thereby advancing core ethical values such as privacy, fair access to datasets, digital divide, or autonomy.

#### Business models for responsible innovation

Participants emphasized the importance of identifying and further developing business models centered around ethical and societal benefits in digital health. At the core, these models should consist of a clear, societally aligned purpose. They should demonstrate a clear focus on societal benefit, and how it translates into tangible competitive advantage. Responsible innovation principles and business models must be financially sustainable and advantageous to be adopted and maintained. To this effect, business models that incorporate societal responsibility might showcase how overcoming known hurdles to access digital health tools can result in added commercial value (e.g., expanded customer base). A concrete example that participants discussed is business models that take digital divide into account: digital divide is the phenomenon that disadvantaged groups face barriers to access (e.g., lack of digital devices, network connection, digital literacy skills) and as a result cannot benefit from digital health in the same way as other stakeholders. Responsible business models try to reduce barriers to access for socially disadvantaged communities by, for example, relying on appropriate design choices.

Here, participants noted the considerable influence of Venture Capital (VC) funds, as they typically fund start-ups’ growth. VCs can foster (or prevent) value-driven innovation in at least three ways: at the funding stage, VCs’ investment choices determine which innovation models are realized. VCs also have a considerable indirect effect, as start-ups aspiring to be funded will aim to satisfy VC investment criteria (i.e., responsible practices will be adapted, if VCs reward these start-ups). Once invested, VCs also take on a significant oversight and advisory role in shaping business models and operational decisions. Participants stressed the need for VCs to actively encourage and fund start-ups that adopt responsible innovation practices.

#### Technological design, toolkits, and technical solutions

Technological design was recognized as another central means to addressing ethical issues, for example, bias in datasets, in digital health. Toolkits and technical solutions, such as open-source repositories of ethically compliant technology solutions or ethics best practices for coding, can enable individual innovators to integrate ethics into their work.

#### Self-reflection

Self-reflection by innovators is seen as a major catalyst to achieve values-driven innovation. Innovators, both at the individual and organization level, review the impact and purpose of an innovation at the outset, and throughout the development and implementation phases. As a senior pharmaceutical executive stressed, innovators should ask themselves whether an innovation delivers real utility to patients and end-users and whether they have integrated an ethical perspective on core issues such as privacy, autonomy, or justice.

#### Building awareness

When asked about the ethical challenges and hurdles to responsible digital health, participants repeatedly pointed to the need for ethical training of entrepreneurs and technical developers of digital health applications. Building awareness around responsible innovation and principles such as privacy, equity or autonomy are critical, as innovators often fail to gauge the societal effects of their technology. Innovators should understand the ethical and societal implications of their business and incorporate wider stakeholder perspectives to define and adopt a societally beneficial vision for their products and services. Participants repeatedly pointed to innovation hubs, start-up incubators, and venture capital investors as agents for raising awareness. Such programs provide insight into how to build a network, engage stakeholders early on, and choose board members or co-founders to optimize diversity and representation.

### The three best practice clusters are interdependent

Participants made extensive references to the strong interdependencies between all three solution clusters. Value-based innovation requires innovators to understand, respect, and design for society’s core needs and ethical concerns. Understanding these needs and concerns is unattainable without direct involvement of core stakeholders. Similarly, the concept of responsive regulation heavily relies on exchanging with innovators and societal actors at large. Both pro-active innovator responsibility and stakeholder co-creation rely on an adapted regulatory environment to incentivize, and legally enable stakeholders to adopt co-creation and proactive responsibility. Stakeholder alignment and co-creation are thus a matter of necessity if digital health innovation is to advance ethically and efficiently.

## Discussion

The insights presented herein provide concrete, practical, and mutually reinforcing best practices for responsible digital health. While the literature is ripe with analyses of the ethical and policy challenges of digital health, our work fills a considerable gap on how to practically enable responsible innovation in digital health. Our participants addressed a broad spectrum of obstacles to responsible digital health innovation as recurrently identified in the literature: the role of stakeholders, regulation and ethics^3,21–25^.

These results dovetail in a complementary manner with our previous work on impediments to digital health innovation in which we had identified regulation, ethics, and stakeholders collaboration as areas of concern^8^. Here, based on a separate analysis, we presented best practices across all the previously identified domains. Such best practices reflect our participants’ specific views on how to address governance impediments. It has to be stressed, however, that other solutions could have emerged and could still be imagined for each of the domains.

Co-creation is increasingly regarded as a major success factor for digital health and artificial intelligence. Carusi et al., for instance, pointed out that development and adoption of medical AI is “as much societal as it is technological”^26^, stressing the need for collaboration between experts to address transparency and build trust. In contrast to our inclusive co-creation concept, however, this approach does not envisage active involvement of wider society or regulators in medical AI development. Beyond only providing one-directional user- feedback, co-creation calls for stakeholders’ active participation throughout the innovation cycle. Similar to notions of “shared responsibility” in patient-led research^27^, or genomic innovation^28^, this concept stresses that diverse stakeholders in digital health have a right to actively shape innovation.

Participants emphasized the importance of aligned vision for co-creation, suggesting developing operating norms and common goals. Such an approach is gaining popularity in AI innovation more broadly. Iason Gabriel, a philosopher at leading AI company DeepMind, emphasized the importance of involving lay publics in value alignment to legitimately identify ethical principles^29^. The World Health Organization has put forward a “Digital Health Strategy” that aims to “advance and apply digital technologies towards the vision of health for all”^30^. Defining and following an aligned vision has also been recommended as an enabler for public-private partnerships^31^.

Our participants’ calls for regulatory innovation and responsive regulation resonate strongly with emerging regulatory practice^32^. In Europe, the European Medicine Agency has begun to assess and adopt the use of artificial intelligence applications in its regulatory practices^33^. In the United States, the Food and Drug Administration (FDA) has established the Digital Health Center of Excellence to lead regulatory innovation. The FDA’s pre-certification program, for instance, was a pilot program to test a novel approach to regulate machine learning applications for medical use. Often relying on unsupervised learning, machine learning applications tend to change continuously. Rather than seeking to certify such rapidly evolving applications at one given point in time prior to market launch, as is common for medical devices, the pre-cert program sought to certify and regulate the developers and their innovation practices behind novel applications^34,35^.

Despite such regulatory forays, the need for legal and operational grounds for responsive regulation remains. In a recent evaluation report of the pre-cert program, the FDA has highlighted that it requires additional legislation and congressional authority to continue as the pre-certification pilot program lacks a firm legal basis^36^. This has re-introduced considerable regulatory uncertainty around responsive regulation among digital health practitioners. The FDA’s report, has, however, strengthened the case for co-creation in responsive regulation: it emphasizes that early and regular inter-stakeholder learning was vital for the FDA to advance regulate novel digital health products and regulatory innovation.

Responsive regulation adapts regulatory measures to the specific characteristics and risk of a given industry or sector. In many countries, some health products and services are subject to formal regulation, while others are not. Non-regulated products could encompass wellness apps, general health information websites, or fitness trackers, which often do not fall under the same regulatory scrutiny. This aggravates the ethical issues common in digital health^37,38^. The need for responsive regulation thus cuts across regulated and non-regulated digital health products. Responsive regulation mechamisms are relevant for non-regulated digital health products because they allow for flexibility and innovation while still ensuring responsible outcomes. Soft law, self-regulation, and experimental law approaches, such as regulatory sandboxes, can effectively fill the governance gap for currently non-regulated products by fostering trust and demonstrating a commitment to ethical practices, ultimately benefiting both consumers and the overall industry.

Despite their potential, however, experimental law, soft law, and self-regulation should be applied cautiously and only in contexts that are adequate for their use. For instance, it has been shown that poor design and implementation of experimental regulation and sandboxes can be problematic or even violate legal principles such as equal treatment and proportionality^39^. Similarly, the OECD has also recently pointed out that while sandboxes are a relevant tool for regulatory learning for fast-moving technology like AI, they are not scalable to all regulatory contexts^40^. Furthermore, challenges around inefficient implementation, the risk of regulatory fragmentation, and lack of technical expertise of regulators should be addressed^40^. Commentators have also pointed out that soft law and self-regulation without capable regulatory oversight can lead to digital innovation that is insufficiently responsible^41^. To mitigate this risk, the EU set up pre-requisites for sandboxing. Innovation must be genuine and there has to be a demonstrable need for testing and risk mitigation. This points to the importance of situating soft law and self-regulation in a wider responsive regulation framework, where regulators are empowered to calibrate their approaches and eventually enforce regulation if innovators fail to fulfil their obligations under soft law and self-regulation.

Many digital health stakeholders identified lack of ethical awareness of practices and principles, as well as training and funding, as key impediments to adopting responsible innovation practices^8^. This finding is in line with Oftedal et al.’s observation that digital health firms have low strategic awareness of responsibility, which translates into “an absence of focused strategies to exercise responsibility”^42^. Oftedal et al. further find that knowledge “on how businesses …[innovate] responsibly is scarce”^7^. Further, even when institutions are aware of responsibility, their lack of resources and funding constitute obstacles to responsible innovation practice^8^. Beyond awareness, it has also been shown that practical translation of ethics into coding and design practices, and business models, is required to ensure that innovation creates real societal value^16^.

Our participants emphasized that VC funds can meaningfully reinforce responsible innovation practices. Indeed, VC funds and researchers have recently made efforts to establish ESG (*short for:* Environmental, Social, and Governance) standards for the VC industry. The VentureESG community, for instance, aims to make ESG “standard part of diligence, portfolio … and fund management”^43^. However, while a practical framework for bio-tech and VC has been published^44^, no comparable framework is available for digital health. Recent literature has further validated our participants’ claim that VCs play an instrumental role in reinforcing corporate responsibility^45^, albeit often a negative one^46–48^. Further participatory research should be conducted with innovators and investors to advance a framework for Venture Capital firms’ role in furthering responsible digital health innovation.

Participants also stressed the importance of translating principles into technological design. There appears to be considerable scope to provide more technological guidance, and concrete examples of incorporating principles into technology development. Indeed, a growing body of literature offers and reflects on responsible design for digital health research^49^, interventions^50–52^, and technology development^11^.

Expecting technology alone to resolve ethical issues and translate ethics into digital health practice, however, constitutes a considerable over-simplification. Several participants exhibited strong optimism in technology and algorithms to “solve ethics”. While these can be a vital translational force, commentators have warned against reducing ethical problems in technology (e.g., privacy) to techno-solutionism, the belief that technology alone will adequately solve major societal and political problems^53^. As stated by Borg^16^ “technical ethical AI tools are necessary, even if insufficient, for addressing the ethical issues AI poses”. Going forward, we expect the need for ethical reflection and responsibility to remain relevant at all levels of technology evolution. Core and auxiliary “ethical technology” will inevitably have blind spots, while new ethical issues will also emerge as technology solves old ones. As such, the need for intensive stakeholder co-creation is likely to remain dominant.

## Methods

### Our wider research project: aims and context

The research presented in this paper is part of a broader research project aiming at identifying innovation and governance best practices for responsible digital health innovation. In this wider project, we aimed to first analyze the stakeholders’ ecosystem that shapes digital health innovation and chart obstacles to responsible innovation, before turning to the identification of innovation and governance best practices. As such, our research project revolved around separate, yet complementary research questions and analyses:

*RQ1: What hinders (responsible) digital health innovation?*

*RQ2: What are innovation and governance best-practices to advance responsible digital health innovation?*

We presented a detailed account of the preliminary study results for RQ1 in a separate study^54^. This paper presents the results of our stakeholder engagement and corresponding analyses for RQ 2.

### Study approach and core research activities

We designed our study to reveal hard-to-access practical knowledge and stakeholders’ attitudes towards digital health innovation and governance. To collect stakeholder views we adapted a mind-mapping methodology called ECOUTER^18^. We chose this participatory methodology over other qualitative methods (e.g., semi-structured interviews, Delphi) because it allowed a high degree of inter-participant exchange thus yielding deeper and richer insights from participants as a group. To promote engagement from a broad spectrum of individuals, ECOUTER typically jumpstarts the mind map with a small set of initial themes, cues, and sub-questions, along with relevant evidence-based references. Participants then tap into their own expertise on related matters to contribute ideas, concepts, evidence, and proposals to the mind map.

We set up the ECOUTER process to allow participants to provide insights in the form of individual comments on four pre-specified sections of the mind-map (see below: Stage I). Participant where free to contribute to any (not necessarily all) sections of the mind-map, as well as to comment on previously posted comments of other participants. Thanks to an unexpectedly high level of stakeholder engagement in the ECOUTER process, we managed to attain sufficient saturation for both research questions. As we describe in detail below, we ensured sufficient distinction between the two research questions by separating data collection (different sections of the mind map), coding (different code books), and thematic analyses which led to two standalone, yet complementary research outcomes.

**Fig. 5:**
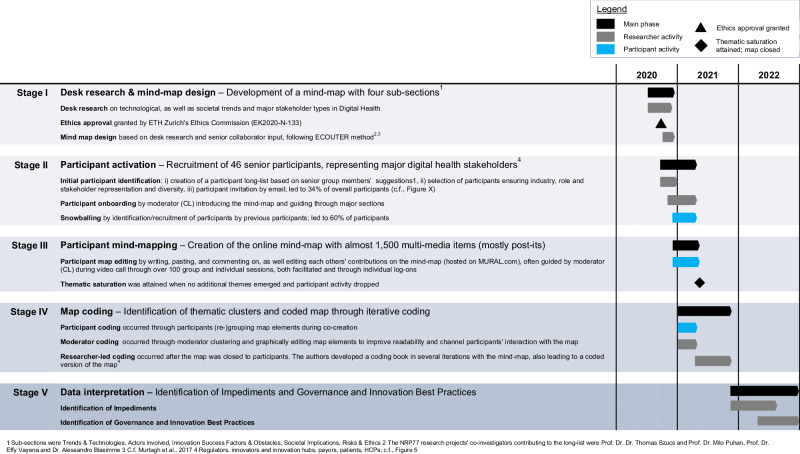
Overview of core research activities across project stages. Gantt chart overview of the major research activities in the course of different project stages. Adapted from ref. ^8^.

As shown in Fig. 5, our methodology for the results presented in this paper followed five steps: I: desk research and mind map design, II: participant activation (recruitment), III: Participant mind-mapping, IV: map coding, and V: data interpretation.

### Stage I: desk research and mind-map design

**Fig. 6:**
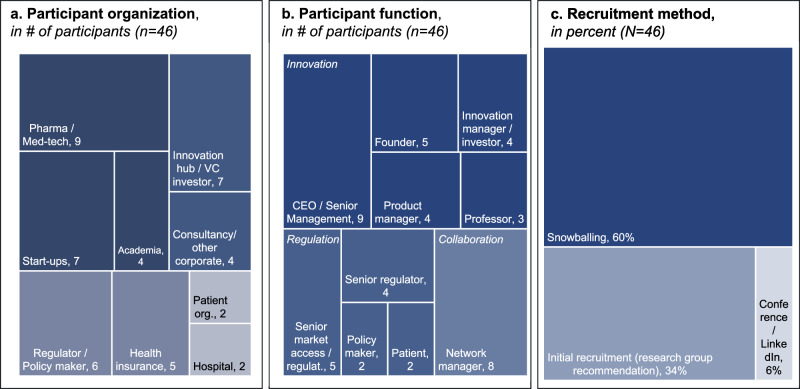
Description of research participants. Tree map illustration of participants’ characteristics. **a** shows research participants’ employment organization (innovator organizations are dark blue, innovation enablers mid blue, regulators bright blue and implementors & end-users grey; compare Table 2 for stakeholder archetypes). **b** details participants function or job role (dark blue corresponds to innovation, mid blue to regulation, bright blue to collaboration and network roles). Panel **c** breaks down participant recruitment method. The overall space of each panel represents the overall participant group (*N* = 46), the area of each subcategory is proportional to its relative share. Adapted from ref. ^8^.

We conducted desk research to gauge technological as well as societal trends and identify the relevant stakeholders in digital health innovation (see Fig. 6) and to isolate key areas to discuss digital health governance, namely: 1) Trends & Technologies; 2) Actors involved; 3) Innovation success factors and obstacles; and 4) societal implications, risks, and ethics. The mind-map was hosted and edited on the online platform MURAL.com. Each area was represented as a dedicated area of a Mural board and included prompts to start off discussion with stakeholders. From the beginning, we maintained a clear distinction between the research questions and their corresponding data. Section 3 of the mind map (“Innovation success factors & obstacles”) was, for example, clearly divided into two subsections with distinct prompts corresponding to the two research questions: *“Core obstacles: what hinders (responsible) digital health innovation?”* and *“Factors of success: what accelerates digital health innovation? What is needed to overcome obstacles?”*. Similarly, section 4 (“Social implications, risks & ethics”) distinguished between a sub-section addressing “*Ethical concerns & risks: What are moral & societal concerns regarding digital health innovation?”* and another *“Means to address ethical concerns: How can moral and societal concerns be integrated with the innovation process?”*.

### Ethics approval

An ethics approval for our research was granted by the ETH Zurich Ethics Commission on October 28, 2020 (EK 2020-N-1330). This research request, as well as an introduction leaflet provided to all participants, clearly highlighted our intention to collect information on both, obstacles, and success factors. Written consent was obtained from all participants.

### Stage II: participant activation

We selected and invited initial participants based on diversity of stakeholder type and role (34% of overall participants). We then used a snowballing approach to recruit the majority of participants (60%) and identified additional participants via LinkedIn (6%). As shown in Fig. 6, our stakeholders represented a broad range of senior digital health stakeholders.

### Stage III: participant co-creation

After participants agreed to participate and submitted a consent form, they were introduced to the mind map by the moderator (CL) during recorded video calls. The moderator asked each stakeholder to comment on the prompts in each thematic area of the Mural board. Each stakeholder was granted access to the Mural board after the moderated session, with the possibility of further editing the mind map by posting additional notes or by commenting on other participants’ notes.

46 stakeholders posted, edited, and discussed our digital mind-map hosted on the web-based platform MURAL.com from December 2021 until April 2022. The map emerged through over 100 sessions of individual editing and facilitated (group) discussions, in which participants added over 1500 notes (virtual post-its) and other mind-map items. Participants frequently returned to the mind-map after their initial contribution, reacting to other participants’ contributions. It is important to highlight that individual participants did not necessarily contribute equally to different parts of the mind-map. Moreover, in the course of the engagement process (5 months), the focus of the participants’ activity—on the mind-map and their engagement with other stakeholders’ input—shifted thematically across sections, with increasing attention to the identification of best practices towards the end of the engagement process. Throughout these activities, the moderator curated the map to increase readability, for instance by grouping thematically similar comments under a given thematic area of the board. Stakeholder engagement was completed after we attained thematic saturation, i.e., when no additional themes emerged, and participant interaction fell.

### Stages IV and V: map coding and data interpretation

The authors performed iterative thematic coding of the mind map. We considered each section of the mind map on its own terms and analyzed it by means of dedicated thematic coding. In this analysis we maintained a clear thematic distinction, conducting two separate coding rounds—one focused on impediments (RQ1) and one on best-practices (RQ2). We devised and then iteratively developed a distinct coding structure for innovation and governance best practices for responsible digital health innovation. A subsequent analysis and interpretation of this coding structure led to the identification of best practices presented above.

### Limitations and future steps

Our research design was focused on making the specific insights of leading digital health practitioners accessible. As such, the insights presented here are not intended to be fully representative. However, the study could have further benefited from additional patient and healthcare practitioner perspectives. The findings and approaches discussed in this paper reflect the practical reality in Switzerland and may differ in other socio-economic and cultural contexts. Biases may have also been introduced through the participant selection process and stakeholders’ extensive interaction among each other.

Going forward, future work should investigate the incentives of individual stakeholders to adopt the outlined best practices and thus identify measures to increase their practical adoption. In the face of fast-moving technological innovation, future research should also monitor and test the effectiveness of the outlined best practices.

## Data Availability

This publication’s raw data (digital mind map edited by participants) cannot be publicly provided due to privacy and confidentiality restrictions. Participating stakeholders cannot be sufficiently anonymized due to the detailed information provided and referenced, as well as the extensive stakeholder interaction that occurred and the technology features of the MURAL platform. Access to raw data is possible on an individual request basis, pending explicit consent of all participants. Requests to access the datasets should be directed to Effy Vayena.
